# Andrographolide Inhibits Rabies Virus Replication

**DOI:** 10.1155/tbed/8934817

**Published:** 2026-09-22

**Authors:** Yue Zhang, Lanxi Zheng, Yueze Wang, Songze Zhao, Hongling He, Shile Huang, Jun Luo, Xiaofeng Guo

**Affiliations:** ^1^ College of Veterinary Medicine, South China Agricultural University, Guangzhou 510642, China, scau.edu.cn; ^2^ Department of Biochemistry and Molecular Biology, Louisiana State University Health Sciences Center, 1501 Kings Highway, Shreveport 71130–3932, Louisiana, USA, lsu.edu; ^3^ Department of Hematology and Oncology, Louisiana State University Health Sciences Center, 1501 Kings Highway, Shreveport 71130–3932, Louisiana, USA, lsu.edu; ^4^ Feist-Weiller Cancer Center, Louisiana State University Health Sciences Center, 1501 Kings Highway, Shreveport 71130–3932, Louisiana, USA, lsu.edu

**Keywords:** andrographolide, antiviral, rabies virus, replication, survival rates

## Abstract

Rabies is a fatal zoonotic disease caused by infection with the rabies virus (RABV). Anti‐RABV serum or antibody is a unique treatment for patients infected by RABV. However, the mortality is almost 100% in the underdeveloped regions due to the high price and the high cost of transportation and preservation of anti‐RABV serum or antibody. Therefore, it is urgently needed to develop novel therapies that can effectively fight against RABV infection. Andrographolide (Andro) is a labdane diterpenoid isolated from *Andrographis paniculate*, which has been used to treat bacterial and viral infections for decades. In this study, we found that Andro dose‐dependently inhibited RABV replication in host cells, with a half‐maximal effective concentration (EC_50_) of slightly less than 60 μmol/L. The antiviral effects of Andro were independent of viral strains and cell lines. Pretreatment of RABV with Andro in test tubes for 1 h did not affect virus replication in host cells, implying that Andro did not directly reduce RABV viability. Of interest, Andro treatment showed no influence on either RABV binding to cells or RABV invasion of cells. Also, Andro treatment downregulated the expression of RABV genomic RNA and structural genes in the cells. To investigate the therapeutic efficacy of Andro in vivo, mice were administered Andro via intraperitoneal (i.p.) injection or intragastric (i.g.) gavage. Neither the administration route nor the dosage significantly affected body weight. Notably, Andro protected mice against a lethal challenge with RABV strain challenge virus standard 11 (CVS‐11). Specifically, i.p. injection of 100 mg/kg and 1 mg/kg Andro increased survival rates from 20% to 60% and 80%, respectively, while i.g. gavage of 250 mg/kg Andro elevated the survival rate from 30% to 60%. Correspondingly, RABV viral loads in mouse brain tissues were also reduced. The results suggest that Andro has great potential as a novel anti‐RABV agent for the treatment of rabies.

## 1. Introduction

Rabies is a virulent zoonotic disease caused by the rabies virus (RABV), which mainly affects the central nervous system of warmblooded animals. Once clinical symptoms appear, almost 100% of the animals die [1]. Although some developed countries in Europe and the United States claimed that human rabies has been eliminated [2], rabies is still a health threat in most developing countries in the world, especially in Asia and Africa [3]. Preexposure prophylaxis of infection (PrEP) is the best way to prevent rabies [4, 5]. In the absence of PrEP, people can prevent rabies through postexposure prophylaxis (PEP). For category III exposure, RABV immunoglobulin G (IgG) can also be administered. In recent years, numerous antibody‐based therapies have been developed for the treatment of RABV infection [6, 7], yet their costs are substantially higher than those of preexposure vaccination. Notably, the majority of global rabies cases occur in less‐developed regions. There are certain difficulties in mass immunization. Therefore, it is difficult to eliminate human rabies on a global scale.

There is currently no specific cure for rabies, although humans have been fighting rabies for centuries [8–11]. Recently, ribavirin (RBV), a guanosine analog that can stop viral RNA synthesis and viral mRNA capping [12], has been found to have anti‐RABV activity [13]. Favipiravir, another nucleoside analog, can inhibit RABV in vitro, but whether it can significantly improve the survival rate of RABV‐challenged mice is controversial [14, 15]. Gamma‐carrageenan has been shown to inhibit RABV internalization and G‐protein‐mediated cell fusion, thereby inhibiting RABV replication [16, 17]. Pyrimethamine inhibits rabies infection in vitro by inhibiting adenosine synthesis [18]. Amantadine, an antidyskinetic medicine, can prevent RABV from entering host cells [19]. In addition, bis‐coumarin derivatives [20], Resveratrol [21], PGG (1,2,3,4, 6‐pentagaloylglucose) [22], and artemisinin derivatives (artesunate and dihydroartemisinin) have also been reported to possess anti‐RABV effects. Nevertheless, none of the abovementioned agents has been approved for the clinical treatment of rabies. Therefore, there is an urgent need to develop new effective therapies for rabies [23].

Andrographolide (Andro) is a labdane diterpenoid derived from the plant *Andrographis paniculate*. It has antibacterial [24], antiviral [25], antiinflammatory [24], and antitumor [26] effects. Andro has been shown to reduce cholestatic liver injury induced by alpha‐naphthalene isothiocyanate in rats [27] and acute brain injury in rats with traumatic brain injury models [28] and alleviate viral myocarditis in mice by inhibiting proinflammatory response [29]. Studies have shown that Andro and its derivatives have a significant antiviral effect [16], which has been demonstrated in HIV [30], Chikungunya virus [31], dengue virus (DENV) [32], influenza virus H1N1 [33], enterovirus EV‐D68 [34], and porcine reproductive and respiratory syndrome virus (PRRSV) [35]. In addition, due to its multiple antiviral activities and relative safety, Andro has also been used as a potential treatment for coronavirus disease (COVID‐19) caused by the novel coronavirus SARS‐CoV‐2 in recent years [36]. However, the effect of Andro on RABV remains unknown.

Here, we show that Andro treatment dose‐dependently inhibited RABV replication. Mechanistically, Andro treatment did not directly reduce RABV viability but interrupted its binding to host cells. Also, Andro treatment downregulated the expression of RABV genomic RNA and structural genes. Our findings suggest that Andro is a potential candidate as an anti‐RABV agent.

## 2. Materials and Methods

### 2.1. Cells, Viruses, Antibodies, and Various Chemical Substances

Mouse neuroblastoma (NA) cells (Wuhan Institute of Biological Products, Wuhan, China) were cultivated in Roswell Park Memorial Institute (RPMI) 1640 medium (Gibco, Suzhou, China), which is a standard growth medium provided by 10% fetal bovine serum (FBS) (Gibco, Suzhou, China). Baby hamster kidney fibroblast (BHK‐21) cells and BSR cells (a clone of baby hamster kidney‐21 cells) (Wuhan Institute of Biological Products, Wuhan, China) were maintained in Dulbecco’s Modified Eagle’s Medium (DMEM) (Gibco, Suzhou, China) supplemented with 10% FBS.

HEP‐Flury (rescued via plasmid pHEP‐3.0, a gift from Dr. Kinjiro Morimoto) and challenge virus standard 11 (CVS‐11, a gift from Dr. Xianzhu Xia, Academy of Military Medical Sciences, Beijing, China) were propagated in NA cells. GD‐SH‐01 is a wt RABV strain that was isolated from the brain tissue of a rabid pig in our laboratory and is phylogenetically close to canine RABV [37]. Fluorescein isothiocyanate (FITC)‐labeled anti‐RABV N antibodies were obtained from Fujirabio Inc. (Malvern, PA, USA). Monoclonal antibodies targeting the M and G proteins of the RABV were self‐prepared and preserved in our laboratory. Andro was acquired from Chengdu Pufei De Biotechnology Co., Ltd. (Chengdu, China), with a purity level of no less than 99.3%. RBV was acquired from Selleck Chemicals (Houston, TX, USA), with a purity level of no less than 99.89%. This compound was solubilized in dimethyl sulfoxide (DMSO, Sigma–Aldrich, St. Louis, MO, USA), and the final concentration of DMSO in the cell culture medium was adjusted to 0.1% (v/v).

### 2.2. Cell Viability Assay

The study evaluated the toxic effects of Andro on both NA and BHK‐21 cell lines by employing the Cell Counting Kit‐8 (CCK‐8) assay. As outlined in the prior protocol [38], the current study was conducted in accordance with the established procedures, incorporating only minor adjustments to the original framework. A series of experiments were conducted by seeding monolayers of either NA cells or the well‐characterized BHK‐21 cell line in cell culture well plates and then exposing these coated substrates to Andro at varying concentrations from 0 to 100 μmol/L for a period of 48 h. Following the administration of the treatment, the CCK‐8 test was conducted to evaluate the effect. To analyze the absorbance of the samples, measurements were taken at a wavelength of 562 nm using a reference value of 620 nm, and these data were collected by an automated microplate spectrophotometer model Ledetect 96 (Labexim Products, Lengau, Austria).

Cell viability percentages were determined by normalizing absorbance values of Andro‐treated groups against those of DMSO‐treated control groups. The 50% cytotoxic concentration (CC_50_) was computed via nonlinear regression analysis (curve fitting) using Prism 6 software (GraphPad Software, CA, USA), with each experimental condition being tested in triplicate. For evaluation of antiviral efficacy, NA or BHK cell monolayers in 96‐well plates were first infected with either RABV HEP‐Flury, CVS‐11, or GD‐SH‐01 at a multiplicity of infection (MOI) of 0.1 for 48 h. Following viral adsorption, the infected cells were treated with Andro at concentrations spanning from 0 to 1000 μmol/L for 48 h. Subsequently, in order to assess the impact of Andro on the virus, we collected the supernatants of RABV‐infected NA cells and BHK‐21 cells treated with different concentrations of Andro for 48 h using the same method as before. We then determined the TCID_50_ of the virus through direct immunofluorescence and calculated the 50% effective concentration (EC_50_) of Andro against RABV.

### 2.3. Virus Titration

Virus titers were quantified through the direct fluorescent antibody (dFA) assay, as described in previous studies [39]. Briefly, NA cells propagated in 96‐well cell culture plates were inoculated with tenfold serial dilutions of the target virus in RPMI 1640 medium and then incubated at 37°C under a 5% CO_2_ condition for 48 h. After incubation, the used culture medium was aspirated, and the cells were fixed with 80% acetone at −20°C for 30 min. Following three rounds of washing with phosphate‐buffered saline (PBS), the cells were incubated with FITC‐labeled anti‐RABV nucleoprotein (N) antibodies (Fujirabio) at 37°C for 1 h. Thereafter, antigen‐positive foci were counted under a fluorescence microscope (AMG, Washington, USA). Virus titers were expressed as focus‐forming units per milliliter (FFU/mL), where one FFU was defined as a single fluorescent focus induced by infectious RABV in NA cells. This detection principle follows the immunofluorescence‐focus method described in the World Organisation for Animal Health (WOAH) Terrestrial Manual [40]. The WOAH manual adopts FFD_50_ (50% focus‐forming dose), a limiting‐dilution endpoint titer, while our study performed direct enumeration of fluorescent foci for FFU calculation [41].

### 2.4. Immunofluorescence Staining

Virus titers were quantified through the dFA assay, as described in previous studies [39]. NA or BHK‐21 cell monolayers cultured in 6‐well plates were infected with RABV HEP‐Flury or CVS‐11 at a MOI of one. After incubation for 1 h at 37°C with 5% CO_2_, the supernatants were removed, and the cells were washed with PBS three times. Next, a fresh medium containing 10% FBS and 60 μmol/L Andro or an equal volume of DMSO was added. The cells were then incubated at 37°C with 5% CO_2_ for 48 h, and the supernatants were harvested. Virus titers of samples were determined in NA cells by dFA as described above. All titrations were carried out in triplicate. Then, the cells were stained with FITC‐labeled anti‐RABV N antibodies (Fujirabio), and DAPI and fluorescent foci were analyzed under a fluorescence microscope (AMG, Washington, USA). ImageJ pairwise fluorescence counts were followed by statistical analysis using GraphPad Prism 6.

### 2.5. Quantitative Real‐Time PCR (qRT‐PCR)

NA cells cultured in 12‐well plates were infected with RABV CVS‐11, HEP‐Flury, and GD‐SH‐01 at a MOI of 1. Following 1 h of incubation at 37°C, the medium was replaced with 1 mL of fresh complete RPMI 1640 medium, and different concentrations of Andro (10, 20, 40, and 60 μmol/L) were added separately. Treatment with DMSO, the vehicle for Andro, was used as a control group, with each treatment performed in triplicate. The cells were subsequently cultured at 37°C in a 5% CO_2_ environment for 48 h. After aspirating the supernatants, the cells were washed once with precooled PBS and lysed using the Magzol reagent (Magen, Guangzhou, China) at the specified time points in accordance with the manufacturer’s protocols. Reverse transcription was conducted using the HiScript II First Strand cDNA Synthesis Kit (Vazyme, Nanjing, China). Each sample was assayed in triplicate with the SYBR Green Master Mix (Vazyme), and reverse transcription quantitative polymerase chain reaction (RT‐qPCR) was carried out using a CFX Connect real‐time system (Bio‐Rad, Hercules, CA, USA). The mRNA levels of N, P, M, G, and L genes were normalized to glyceraldehyde‐3‐phosphate dehydrogenase (GAPDH). The primers employed in this study were identical to those reported previously [42], and are presented in Table 1.

**Table 1:** Primers used for qPCR identification.

Gene	Forward primer (5^′^–3^′^)	Reverse primer (5^′^–3^′^)
CVS‐11N	TCACCGCAAGGGAAGCA	AACGGAAGTGGATGAAATAAGAG
CVS‐11P	CGTACCTGGATAATGTTGGAGTC	TTGGAGGGTTAGGAAAGTTGA
CVS‐11M	CTCCGTATGACGATGACCTG	CATTTGGGCTACACGCTTT
CVS‐11G	ATGCCCGAGAATCCGAGAC	CATCCACAAAGCCGCAAGT
CVS‐11L	CCCTCATTACCTACCAGTCTCA	ACCCAGCACTTGCCTCAT
CVS‐11 genome	AGAAGAAAAAGACAGCGTCAATTG	AGAGACCACCTGATTATTGACTTTGA
HEP‐Flury N	TTTAGTCGGTCTTCTCCTGAGTCT	AATCTGCTCTATTCTATCCGCAATGT
HEP‐Flury P	GAGTCCAAATAGTCAGACAAATGA	AGGAAAGTTGACCGAGACATAGGA
HEP‐Flury M	AGAGGACAAAGACTCTTCTCTGCT	TGGAGTTAAGCCCGTATGTTCTCT
HEP‐Flury G	GCCTTGATTGCCCTGATGTTGATAA	CATTTCTCCCTGTCCCTCCAAGAT
HEP‐Flury L	TGTTGATGTCTGATTTCGCATTGTCT	AAGGGAACGCTCTTGACAGATGT
HEP‐Flury genome	AGAAGAAGCAGACATCGTCAGTTG	GGAGACCACCTGATTATTGACTTTGA
GD‐SH‐01N	TACTCATCAAATGCGGTTGGTCAC	GCACATGCGGCAATAACTGTTG
GD‐SH‐01P	CAGGTGTGACTCGTTTAGCTCATG	TGTCTGGCTCAACTAATAGCTGGA
GD‐SH‐01M	TCCAAGGTAGGGTATGGTGTATCAA	AAGAGTCCTTGTCCTCCTCTGAC
GD‐SH‐01G	ACGATAAATCCCTTCATTCGAGAGT	ATGGTGTAGTCATGGTTAGTAGAGC
GD‐SH‐01L	GAGTCTGTCGTCTCACTGGATCA	GATGCCTTACCACCTCTCCAGATA
GD‐SH‐01 genome	AGAAGAAGCAGACATCGTCAGTTG	GGAGACCACCTGgATTATTGACTTTGA
GAPDH	CGTCCCGTAGACAAAATGGT	TTGATGGCAACAATCTCCAC

### 2.6. Western Blot Analysis

NA and BHK‐21 cells were separately seeded into 6‐well plates and cultured until confluent monolayers were formed. Monolayers of NA or BHK‐21 cells were then infected with RABV strains CVS‐11, HEP‐Flury, or GD‐SH‐01 at a MOI of 1. After incubation for 1 h at 37°C with 5% CO_2_ to allow viral adsorption, the inoculum was removed, and fresh culture medium supplemented with 60 μmol/L Andro or an equal volume of DMSO (served as a negative control) was added to each well. At 24 or 36 h postinfection (hpi), the culture medium was discarded, and the cells were washed twice with precooled PBS. Subsequently, the cells were lysed with ice‐cold lysis buffer containing 1 mmol/L phenylmethylsulfonyl fluoride (PMSF) (Beyotime, Haimen, China) to inhibit protein degradation. Following centrifugation at 10,000 ×*g* for 10 min at 4°C, the resulting supernatant was carefully collected. The total protein concentration in each sample was quantified using a microspectrophotometer, which provided precise measurements. A consistent amount of protein (30 μg per sample) was subjected to electrophoresis on 12% sodium dodecyl SDS‐PAGE gels, followed by electroblotting onto polyvinylidene fluoride (PVDF) (Millipore, MA, USA) membranes from a commercial supplier. The membrane‐bound proteins were investigated using the RABV G‐protein antibodies in accordance with the established protocol outlined in the study by Luo et al. [43].

### 2.7. Detection of the Interaction Between RABV and Andro

To investigate whether Andro directly interacts with RABV, RABV (MOI = 1) was mixed with different concentrations of Andro and incubated for 1 h at 37°C in test tubes. RABV and Andro were separated by ultrafiltration centrifugation. Briefly, a mixture of RABV and Andro was added to an ultrafiltration unit (0.5 mL, 30 kDa cutoff) (Millipore, MA, USA), followed by centrifugation at 4°C (7500 g, 10 min). RABV pellets retained in ultrafiltration tubes were washed twice with the basal medium to remove the residual compound and then resuspended in the basal medium to infect NA cells grown in 6‐well plates. After washing three times with PBS, the cells were cultured in fresh medium for an additional 48 h at 37°C. Finally, samples were collected to determine viral titers by terminal dilution, RABV‐infected cell numbers by immunofluorescence assay (IFA), and viral genomic RNA levels by RT‐qPCR.

### 2.8. Assay of the Effect of Andro on RABV Adsorption and Cell Invasion

To evaluate RABV binding to host cells, RABV strains HEP‐Flury or CVS‐11 were thoroughly mixed with 60 μmol/L Andro or an equivalent volume of DMSO (vehicle control). NA cells cultured in 6‐well plates were inoculated with CVS‐11 at an MOI of five and incubated at 4°C for 2 h to allow viral attachment. After aspirating the culture supernatants, the cells were washed three times with PBS to remove the unbound virus particles. Subsequently, 1 mL of Magzol lysate was added to each well, and the lysate was incubated at room temperature for 2 min to achieve complete cell lysis. The resulting cell lysates were transferred to 1.5 mL ribonuclease‐free centrifuge tubes. Total RNA was extracted from the lysates, and the level of viral genomic RNA within the cells (i.e., the quantity of RABV bound to cells) was detected by RT‐qPCR, as described previously [44].

For the assessment of viral invasion, NA cells cultured in 6‐well plates were infected with CVS‐11 at an MOI of five. Following 2 h of adsorption at 4°C, the culture supernatants were discarded, and the cells were washed three times with PBS to remove unbound free virus. Each well was then replenished with 2 mL of fresh complete RPMI 1640 medium supplemented with 60 μmol/L Andro, followed by incubation at 37°C for 2 h to facilitate viral entry. The spent medium was aspirated, and the cells were washed once with PBS. The cells were subsequently treated with 0.25% EDTA‐free trypsin at room temperature for 2 min to remove RABV particles remaining on the cell membrane surface. Afterward, 1 mL of Magzol lysate was added to each well, and the lysate was incubated at room temperature for 2 min to lyse the cells. The resulting lysates were transferred to 1.5 mL ribonuclease‐free centrifuge tubes. Total RNA was extracted, and the viral genomic RNA level in the cells (i.e., the quantity of the invading virus) was determined by RT‐qPCR, as reported previously [45].

### 2.9. Animal Experiment

Select 6–7‐week‐old KM mice and inoculate them with 1.0 × 10^5.5^ FFU of CVS‐11 virus via intramuscular injection [45]. About 2 h after the inoculation, Andro or normal saline was given as the control group by intraperitoneal (i.p.) injection. The i.p. administration dose are as follows: 250 mg/kg for the high‐dose group, 100 mg/kg for the medium‐dose group, and 1 mg/kg for the low‐dose group. Each group should have no fewer than 6 mice. The intragastric (i.g.) administration dose are as follows: 500 mg/kg for the high‐dose group, 250 mg/kg for the medium‐dose group, and 10 mg/kg for the low‐dose group [46]. Each group should have no fewer than 10 mice. The mental state and survival rate of the mice were recorded every day for 21 consecutive days [47]. At 10 days postinoculation, partial mice were anesthetized and subjected to cardiac perfusion with sterile PBS to eliminate residual blood from the brain tissues. Whole‐brain tissues were rapidly dissected and homogenized. Total RNA was extracted from brain homogenates, and RABV viral loads were quantified by RT‐qPCR.

### 2.10. Statistical Analysis

All experimental procedures were conducted in a repeated manner, with each trial being executed in at least three separate instances to ensure reliability and minimize the risk of anomalies. The findings are summarized in the form of average values accompanied by their standard deviations. When evaluating data from two distinct groups, the statistical significance was assessed through a Student’s *t*‐test, whereas for multiple groups, an analysis of variance (ANOVA) was employed to determine significance. When more than two groups were compared.  ^∗^ *p* < 0.05,  ^∗∗^ *p* < 0.01, and  ^∗∗∗^ *p* < 0.001 were considered to be statistically significant at different levels.

## 3. Results

### 3.1. Andro Reduces the Cell Viability in a Concentration Dependent Manner

**Figure 1:**
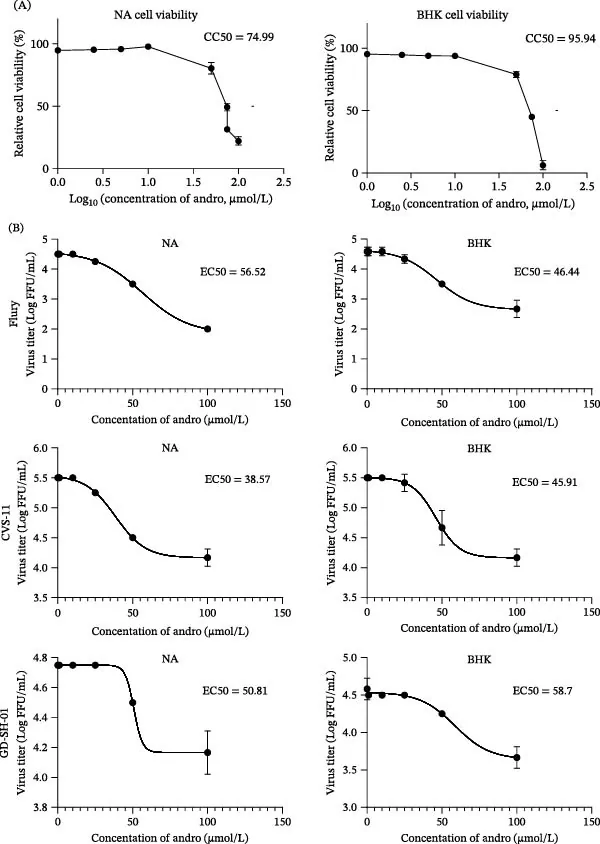
The effects of Andro on the survival of NA cells and BHK cells and its inhibition of RABV. (A) Effects of Andro on the viability of NA and BHK cells. NA or BHK cell monolayers cultured in 96‐well plates were treated with Andro (0–1000 μmol/L) for 48 h, followed by a cell viability assay using the CCK‐8 assay kit. Cells treated with DMSO served as a control. Shown are the mean values from three independent experiments. (B) Effects of Andro on RABV activity in NA and BHK cells. NA or BHK cell monolayers cultured in 96‐well plates were infected with RABV HEP‐Flury, CVS‐11, or GD‐SH‐01 at a MOI of 0.1 for 48 h, and the cell monolayers were then treated with 0~100 μmol/L of Andro for 48 h. Subsequently, culture supernatants were collected and subjected to viral titer determination for RABV. Each experiment was repeated independently three times.

To study the effect of Andro on RABV replication, mouse NA and BHK‐21 cells were employed. To find what concentration of Andro is good for our experiments, the CCK‐8 assay was utilized to determine the cytotoxicity of Andro in the two cell lines. As shown in Figure 1A, 48‐h treatment with Andro decreased the viability of NA and BHK‐21 cells in a concentration‐dependent manner. The calculated 50% of cytotoxicity (CC_50_) values for Andro were ~74.99 μmol/L in NA cells and 95.94 μmol/L in BHK‐21 cells, respectively. The cell viability was not remarkably reduced when treated with Andro at concentrations of 10–60 μmol/L. Hence, Andro was used at these concentrations for all the following experiments.

### 3.2. Andro Inhibits RABV Replication in NA and BHK‐21 Cells

Subsequently, we assessed the impact of Andro on the replication of the attenuated RABV strain HEP‐Flury, the pathogenic RABV strain CVS‐11, and the wild‐type RABV strain GD‐SH‐01 in NA and BHK‐21 cells. As illustrated in Figure 1B, Andro treatment suppressed RABV replication in both cell lines in a dose‐dependent manner. The half‐maximal effective concentrations (EC_50_) of Andro for inhibiting HEP‐Flury infection were 56.52 μmol/L in NA cells and 46.44 μmol/L in BHK‐21 cells, respectively. For CVS‐11 infection, the EC_50_ values of Andro were 38.57 μmol/L in NA cells and 45.91 μmol/L in BHK‐21 cells. Meanwhile, the EC_50_ values of Andro against GD‐SH‐01 infection were 50.91 μmol/L in NA cells and 58.7 μmol/L in BHK‐21 cells.

**Figure 2:**
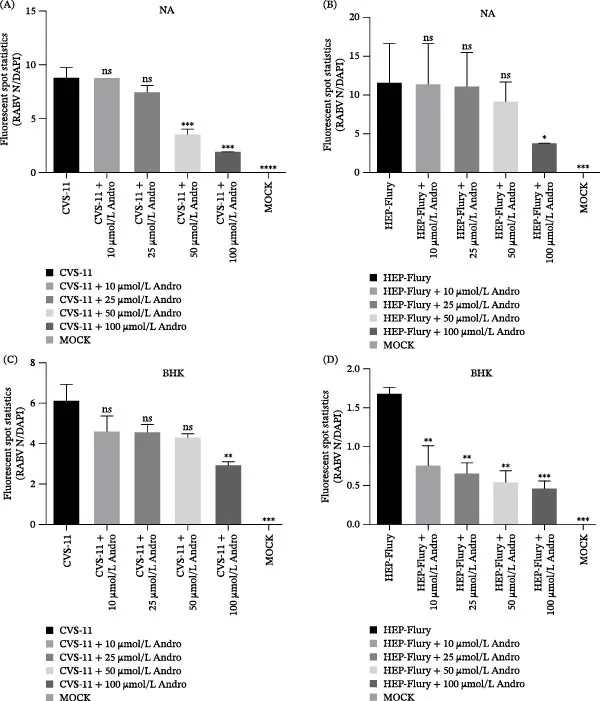
Immunofluorescence assay of Andro inhibiting RABV. NA or BHK‐21 monolayers cultured in 6‐well plates were infected with RABV HEP‐Flury or CVS‐11 at MOI = 1. After incubation for 1 h at 37°C, fresh medium containing Andro (0, 10, 25, and 50 μmol/L) or an equal volume of DMSO was added. The cells were incubated at 37°C for 48 h, and the supernatant was collected. Viral titers of samples were determined by dFA in NA cells. All titrations were performed in triplicate. Cells were stained with FITC‐labeled anti‐RABV N antibody and DAPI, and fluorescent foci were visualized on a fluorescence microscope. Semiquantitative fluorescence analysis at the single‑cell level was performed as previously described [48]. Briefly, DAPI‑stained nuclei were used to define individual cells, and the fluorescence intensity of RABV N protein was quantified for infected cells under identical image acquisition parameters across all groups. A fixed viral MOI was applied for all immunofluorescence experiments, and we focused on the abundantly expressed cytoplasmic RABV N protein for signal quantification. ImageJ pairwise fluorescence counts were followed by statistical analysis using GraphPad Prism 6. (A) Inhibitory effect of Andro on CVS‐11 in NA cells; (B) Inhibitory effect of Andro on HEP‐Flury in NA cells; (C) Inhibitory effect of Andro on CVS‐11 in BHK cells; and (D) Inhibitory effect of Andro on HEP‐Flury in BHK cells.

**Figure 3:**
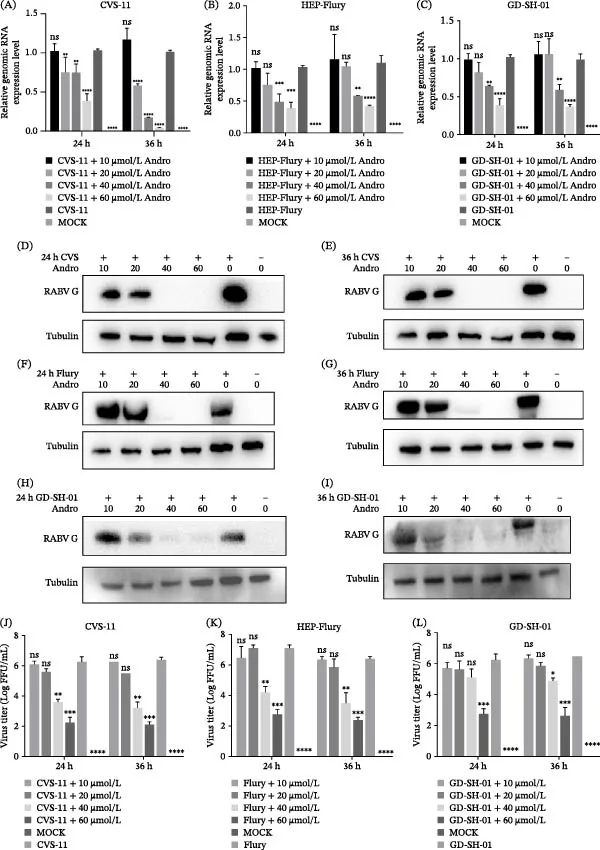
Effect of Andro on RABV replication in NA cells. (A–C) RT‐qPCR analysis of the inhibitory effect of Andro on RABV replication in NA cells. NA cells cultured in 12‐well plates were infected with RABV CVS‐11, HEP‐Flury, and GD‐SH‐01 at an MOI of 1. Each group had three replicate wells. After incubation at 37°C for 1 h, 1 mL of fresh complete RPMI 1640 medium with different concentrations of Andro (0, 10, 20, 40, and 60 μmol/L) was replaced. Cultured at 37°C for 24 h and 36 h. After cells were lysed with Magzol, RNA was extracted and reverse converted into cDNA. The mRNA expression levels of RABV genomic RNA and structural proteins N, P, M, G, and L were detected by RT‐qPCR. (D–I) Inhibition of RABV by Andro on NA cells by Western blot. Cell lysates were prepared from RABV‐infected (CVS‐11, HEP‐Flury, or GD‐SH‐01) and noninfected NA cells treated with different concentrations of Andro. After lysis and centrifugation at 4°C, the supernatant was collected, and total protein concentrations were quantified. Equal amounts of total protein (30 μg/sample) were separated by SDS‑PAGE gel and transferred to PVDF membrane. RABV G‐protein expression was determined by Western blot. (J–L) Inhibition of RABV by Andro in NA cells by viral titer assay. The RABV CVS‐11, HEP‐Flury, or GD‐SH‐01 infected and uninfected NA cells treated with different concentrations of Andro were incubated for 24 and 36 h. The cell culture supernatants were collected after incubation, and viral titers were determined by TCID_50_ assay.

To validate the inhibitory effect of Andro on RABV replication, NA cells and/or BHK‐21 cells infected by CVS‐11 or HEP‐Flury were exposed to different concentrations of Andro or DMSO (control), followed by immunofluorescence staining, RT‐qPCR, and Western blot analysis. As shown in Figure 2 and Figure S1, immunofluorescence demonstrated that the number of RABV‐positive fluorescent spots was markedly reduced in Andro‐treated groups relative to the DMSO‐treated virus‐infected control groups, indicating that Andro could suppress RABV replication. High‐dose Andro significantly diminished fluorescent spots induced by CVS‐11 infection in both NA and BHK‐21 cells (Figure 2A,C). For HEP‐Flury infection, Andro also exerted prominent dose‐dependent antiviral inhibition (Figure 2B,D). Collectively, these results suggest that the anti‐RABV activity of Andro against CVS‐11 and HEP‐Flury is dose‐dependent in NA and BHK‐21 cells. By RT‐qPCR, treatments with higher concentrations of Andro showed better inhibitory effects on the expression of RABV genomic RNAs of CVS‐11, HEP‐Flury, and GD‐SH‐01 in NA cells compared to treatments with lower concentrations of Andro at 24 or 36 hpi (Figure 3A–C). By Western blot analysis, Andro treatment also dose‐dependently reduced the RABV G‐protein level in NA cells at 24 and 36 hpi (Figure 3D–I). By viral titer assay, Andro similarly reduced the production of infectious RABV particles in a dose‐dependent manner between 24 and 36 hpi (Figure 3J–L). Notably, the inhibitory potency of Andro against GD‐SH‐01 was slightly weaker relative to CVS‐11 and HEP‐Flury, which may be related to the distinct genetic background and replication characteristics of this wild‐type strain compared with laboratory‐adapted strains. Nevertheless, significant suppression was still observed for GD‐SH‐01 upon Andro treatment relative to the virus‐only control. Collectively, these results support that Andro inhibits RABV replication in a dose‐dependent manner in host cells.

### 3.3. Andro Does Not Directly Reduce RABV Viability

**Figure 4:**
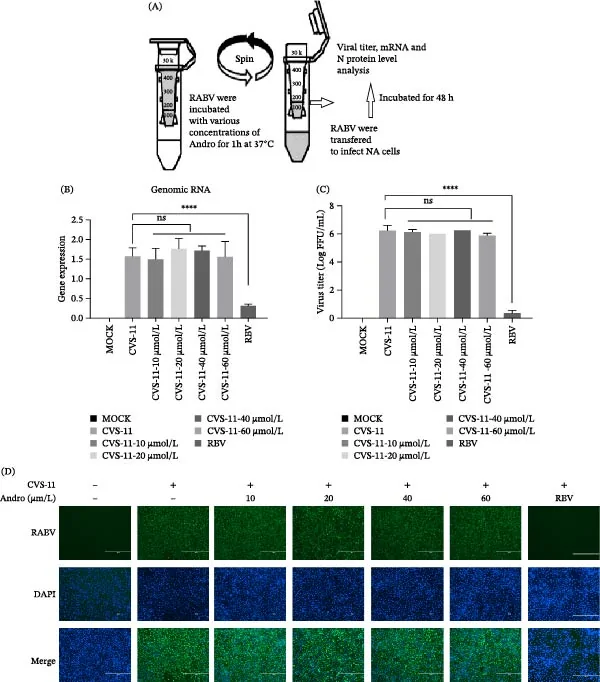
The infection of NA cells with Andro was not affected by Andro pretreatment with RABV. RABV (MOI = 1) was mixed with different concentrations of Andro and incubated for 1 h at 37°C. RABV and Andro were separated by ultrafiltration centrifugation. The remaining RABV particles in the ultrafiltration tube were resuspended in basal medium to infect NA cells grown in 6‐well plates and cultured for 48 h. Samples were collected, and viral titers were detected by the gradient dilution method. RABV‐infected cells were detected by IFA, and viral genomic RNA level was detected by RT‐PCR. (A) Schematic representation of the ultrafiltration process of RABV following incubation with Andro at 37°C for 1 h. (B) Virus titers determined after ultracentrifugation. (C) Analysis of viral genomic RNA post ultracentrifugation. (D) Results of immunofluorescence detection subsequent to ultracentrifugation.

To determine whether Andro directly reduces the viability of RABV, CVS‐11 (MOI = 1) was incubated with different concentrations of Andro (0–60 μmol/L) at 37°C for 1 h. A positive control using 50 mM RBV was included [13]. followed by ultracentrifugation and inoculation of NA cells with the virus according to the procedure shown in Figure 4A. In addition, there was no significant change in the level of RABV viral genomic RNA (Figure 4B) At 48 hpi, there was no significant difference in the titer of RABV pretreated with and without Andro (Figure 4C) or RABV N protein immunofluorescence staining (Figure 4D) in the cells infected with Andro‐pretreated or nontreated RABV. These observations suggest that pretreatment of RABV with Andro does not affect the viability of RABV.

### 3.4. Andro Interrupts RABV Binding to Cells but Does not Affect RABV Invasion of Cells

Intracellular replication of RABV is not only related to the virus itself but also depends on the adsorption and invasion of the virus on the surface of the host cell (internalization) [49]. To study whether Andro inhibits RABV replication by affecting its binding or invading cells, 60 μmol/L Andro was added to NA, followed by infection with RABV at different times. RT‐qPCR was used to detect the genomic RNA level of RABV after binding or invading cells. As shown in Figure 5A, Andro treatment significantly decreased the genomic RNA amount of CVS‐11 binding on NA cells and BHK‐21 cells compared to nontreatment (control). However, Andro treatment did not significantly affect the genomic RNA level of RABV in both NA cells, as detected by the invading assay (Figure 5B). These results indicate that Andro interferes with RABV binding to cells but does not affect RABV invasion of cells.

Figure 5RT‐qPCR analysis of the effect of Andro on the replication cycle of HEP‐Flury cells. NA cells cultured in 6‐well plates were completely mixed with 60 μmol/L Andro or an equal volume of DMSO and RABV HEP‐Flury or CVS‐11 (MOI = 5), incubated for 2 h at 4°C, and washed three times with PBS. After lysis, total RNA was extracted from the cells, and viral genomic RNA in the cells was detected by RT‐qPCR. Similarly, NA cells were infected with RABV HEP‐Flury or CVS‐11, both at a multiplicity of infection of five. After adsorption for 2 h at 4°C, the culture supernatant was discarded. After washing the cells three times with PBS, each well was replaced with fresh complete medium containing 60 μmol/L Andro. After incubation for 2 h at 37°C, the cells were treated with 0.25% trypsin without EDTA for 2 min. RABV on the cell membrane surface was removed, total RNA was extracted after lysis of cells, and viral genomic RNA in cells was detected by RT‐qPCR. (A) Binding assay of CVS‐11 to cells treated with andrographolide; (B) entry assay of CVS‐11 to cells treated with Andrographolide; (C) inhibitory effect of andrographolide on genomic RNA and structural protein synthesis in NA cells at 24 h; (D) inhibitory effect of Andrographolide on genomic RNA and structural protein synthesis in NA cells at 48 h. Data are presented as mean ± SEM (*n* = 3 per group). *p* < 0.05, *p* < 0.01, and *p* < 0.001 compared with the control group.

**Figure:**
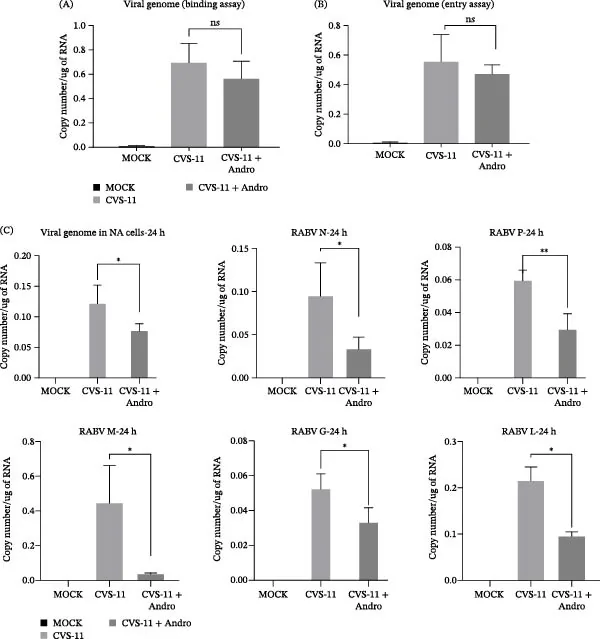

**Figure:**
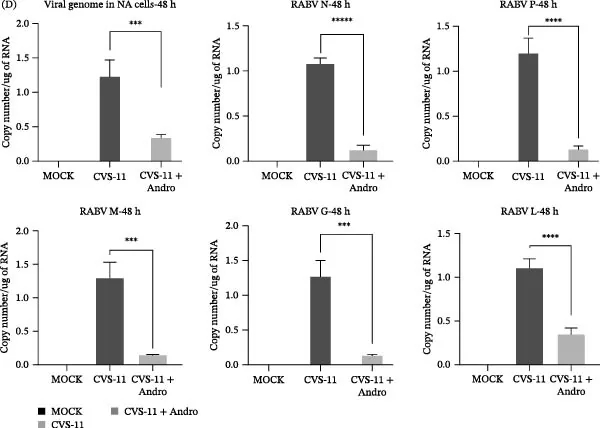

### 3.5. Andro Suppresses the Expression of RABV Genomic RNA and Structural Genes in Cells

After RABV invades cells, the expression (synthesis) of viral genomic RNA and structural genes using raw materials from host cells is essential for the replication of the virus [49]. We hypothesized that Andro inhibits RABV replication by suppressing the expression of its viral genomic RNA and structural genes. To test this, RT‐qPCR was used to detect the levels of genomic RNA and structural gene mRNAs in the cells infected with HEP‐Flury and treated with Andro for 48 h. We found that the expression levels of viral genomic RNA and RABV structural genes (N, P, M, G, and L) in NA cells treated with Andro for 24 and 48 h were significantly lower than those in the control cells (Figure 5C,D). These data demonstrate that Andro suppresses the transcription of RABV genomic RNA and its structural genes.

### 3.6. Andro Protects Mice From Lethal RABV Infection

**Figure 6:**
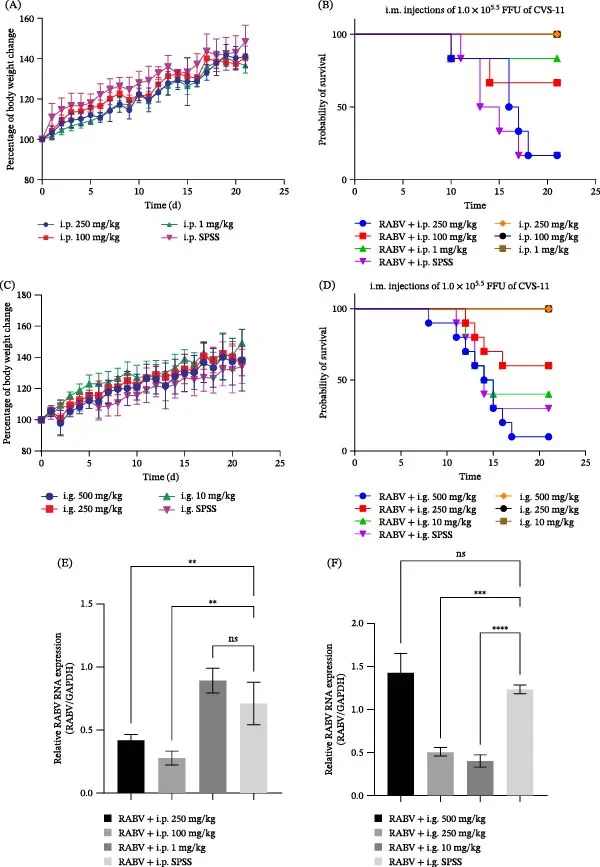
Therapeutic efficacy of Andrographolide in mice administered via different routes. Mice were intramuscularly injected with 100 µL of 1.0 × 10^5.5^ FFU of the highly virulent rabies virus strain CVS‐11 into the gastrocnemius muscle. About 2 h of postinfection, mice were administered different concentrations of Andro or 1% DMSO‐containing saline via intraperitoneal (i.p.) injection or intragastric (i.g.) administration. Noninfected control groups receiving different drug concentrations were also established. Body weight changes and mortality rates were observed in mice for 21 days. The i.p. administration concentrations were high dose (250 mg/kg), medium dose (100 mg/kg), and low dose (1 mg/kg), with 6 mice in each group. While the i.g. administration concentrations were high dose (500 mg/kg), medium dose (250 mg/kg), and low dose (10 mg/kg), with 10 mice in each group. At day 10 postinfection, brain tissues were harvested from mice, and viral loads in brain tissues were quantified by RT‑qPCR. (A, B) Effects of different concentrations of Andro administered by intraperitoneal injection on (A) body weight and (B) survival rate of RABV‐infected mice. (C, D) Effects of different concentrations of Andro administered by gavage on (C) body weight and (D) survival rate of RABV‐infected mice. (E) Detection of RABV virus load in the brain tissue of mice administered intraperitoneally. (F) Detection of RABV virus load in the brain tissue of mice administered by gavage.

To evaluate the potential toxic and side effects of Andro as a drug in mice, administrations were performed via i.p. and i.g. routes, with physiological saline serving as the control. Changes in the body weight of the mice were monitored and recorded. As depicted in Figure 6A,C, neither different administration routes nor drug doses exerted a significant impact on the body weight of mice.

To investigate the therapeutic effect of Andro on RABV‐infected mice, a lethal dose of 100 µL containing 1.0 × 10^5.5^ FFU of the RABV strain CVS‐11 was administered intramuscularly. 2 hpi, different concentrations of Andro were delivered via i.p. and i.g. routes, respectively. As shown in Figure 6B,D, i.p. administration of medium and low concentrations of Andro elevated the survival rate of mice from 20% to 60% and 80%, respectively; in contrast, i.g. administration of medium and low concentrations of Andro increased the survival rate from 30% to 60% and 40%, respectively.

To further evaluate the viral burden in the central nervous system, mouse brain tissues perfused with PBS were collected at day 10 post‐CVS‐11 challenge, and the relative RABV RNA expression in brain tissues was determined by RT‐qPCR (Figure 6E, i.p. groups; Figure 6F, i.g. groups). Under i.p. administration, both 250 and 100 mg/kg Andro significantly reduced brain RABV RNA levels, whereas no significant difference in cerebral viral RNA was observed in the low‐dose 1 mg/kg group compared with the solvent control (ns). For i.g. treatment, 250 and 10 mg/kg Andro markedly downregulated brain RABV RNA, while no obvious reduction of viral RNA was found in the highest‐dose 500 mg/kg group (ns). These results indicate a dissociation between brain viral RNA levels and in vivo protective efficacy, and suppression of cerebral viral replication alone is insufficient to achieve optimal survival against lethal RABV infection.

## 4. Discussion

Andro and its derivatives have been shown to possess potent antiviral activity against HIV [30], Chikungunya virus [31], DENV [32], influenza virus H1N1 [33], enterovirus EV‐D68 [34], and PRRSV [35]. However, whether Andro can inhibit RABV remains unclear. Here, we present evidence that Andro dose‐dependently inhibits RABV replication. This is partly related to its ability to downregulate the expression of RABV genomic RNA and structural genes in host cells.

Andro, a natural product extracted from the plant *Andrographis paniculate*, has been approved and widely used for liver protection [50], antibacterial, antiinflammation [51], heat relief, and detoxification [52] in China. Andro is a promising drug for repurposed use as it has a good safety profile clinically [24]. In addition, Andro is a diterpenoid lactone compound, which can pass through the blood‐brain barrier (BBB) and distribute to different brain regions [53]. Thus, its pharmacological effects on the central nervous system have begun to be revealed in recent years. For example, studies have reported that Andro can reduce the volume of cerebral infarction in several cerebral ischemia models [54]. Andro can also mitigate acute brain injury in a rat model of traumatic brain injury [28]. These previous studies provide a better foundation for us to investigate the inhibitory effect of Andro on RABV in vivo in future research.

Previous studies have described multiple mechanisms of action of anti‐RABV agents. For instance, favipiravir, which is a purine nucleic acid analog and a novel broad‐spectrum anti‐RNA viral drug, can inhibit RABV in vitro and significantly improve the survival rate of RABV‐challenged mice in vivo by inhibiting viral RNA polymerase [15]. Pyrimethamine inhibits RABV replication by inhibiting adenylate synthesis [18]. Gamma‐carrageenan has been shown to inhibit RABV internalization and G‐protein‐mediated cell fusion, thereby inhibiting RABV replication [16]. In this study, we observed that pretreatment with 60 μmol/L Andro for 30–60 min in vitro did not affect the viral replication in host cells, implying that Andro did not reduce the viability of RABV. In addition, we found that inhibiting the expression of both viral RNA and proteins also contributed to the anti‐RABV activity of Andro. A new question that arises is how treatment with Andro profoundly reduces the levels of RABV genomic RNA and structural gene mRNAs. A new question is how treatment with Andro profoundly reduced the levels of genomic RNA and structural genes (mRNAs) of RABV. The RNA level is known to be regulated at transcription (synthesis) and posttranscription (degradation) levels [44]. Further research is needed to determine whether Andro downregulates the expression of genomic RNA and structural genes of RABV by inhibiting their synthesis and/or promoting their degradation.

In this study, Andro demonstrated significant therapeutic efficacy against RABV infection in mice, particularly at optimal concentrations. i.p. administration of medium (100 mg/kg) and low (1 mg/kg) doses of Andro increased survival rates from 20% to 60% and 80%, respectively, while i.g. delivery of medium (250 mg/kg) and low (10 mg/kg) doses improved survival from 30% to 60% and 40%, respectively. Notably, our RT‐qPCR results from brain tissues further revealed a dissociation between cerebral viral RNA levels and the survival outcome. Although the medium–high i.p. doses (100 and 250 mg/kg) effectively reduced brain RABV RNA; this viral suppression did not translate into maximal survival benefit. Strikingly, the 1 mg/kg i.p. group achieved the highest survival rate without significant reduction of brain viral load. A similar inconsistency was observed in i.g. groups: 10 and 250 mg/kg Andro markedly decreased brain RABV RNA, whereas the highest 500 mg/kg i.g. group lost the capacity to suppress viral replication in the central nervous system and conferred almost no survival protection. Conversely, higher doses of Andro paradoxically increased mortality, with survival rates of 20% (250 mg/kg, i.p.) and 10% (500 mg/kg, i.g.). This effect is unlikely attributable to direct toxicity as neither dosing regimen nor concentration significantly affected body weight. Collectively, these observations suggest that the in vivo protective activity of Andro cannot be explained solely by its ability to lower viral RNA burden in the brain. Mechanistically, certain antiviral agents (e.g., RBV) may suppress Th1‐type immune responses [55], impairing viral clearance. High drug concentrations could further compromise immune cell function, facilitating viral dissemination in the central nervous system. Additionally, while some drugs interfere with viral entry pathways, supratherapeutic doses might inadvertently promote alternative routes of infection (e.g., cell‐to‐cell fusion). At low to medium concentrations, the drug can exert a protective effect by regulating host immunity and targeting key viral proteins. However, when the dose is excessively high, it may disrupt the body’s normal physiological balance (such as immune homeostasis and cellular metabolism), which not only offsets the antiviral effect but may even cause adverse impacts. This finding provides a core reference for subsequent formulation development and clinical dose setting, enabling a balance between “efficacy” and “safety” through precise dose control. For instance, adamantane derivatives inhibit viral entry at low concentrations but destabilize cell membranes at high doses, accelerating viral release [56, 57]. These findings underscore the critical need for dose optimization, necessitating pharmacokinetic (PK) and pharmacodynamic (PD) studies to define a safe and effective therapeutic window. For example, favipiravir requires 2–3 times higher doses to inhibit CNS viral replication in murine models, yet excessive dosing induces toxicity [58]. Despite these challenges, appropriately dosed Andro effectively protected mice against a lethal RABV challenge, supporting its potential as a novel candidate for rabies treatment.

In summary, this study shows that Andro exerts a strong inhibitory effect on RABV replication in the cell culture. Mechanistically, this effect is attributed to the downregulation of RABV genomic RNA and structural gene expression in host cells. Moreover, Andro exhibits significant therapeutic efficacy against RABV in a mouse model. Given that Andro is well‐tolerated and commercially available, it holds great potential for development as an anti‐RABV agent.

## Author Contributions

Conceptualization: Yue Zhang, Xiaofeng Guo, and Jun Luo. Methodology: Jun Luo, Yue Zhang, and Hongling He. Investigation: Yue Zhang, Lanxi Zheng, and Yueze Wang. Writing: Yue Zhang, Shile Huang, and Jun Luo. Software: Songze Zhao. Funding acquisition: Xiaofeng Guo and Jun Luo. Supervision: Xiaofeng Guo, Jun Luo, and Shile Huang.

## Funding

This study was partially supported by the National Key Research and Development Plan (Grant 2022YFD1800100), the National Nature Science Foundation of China (Grant 32473115), and the Specific University Discipline Construction Project (Grant 2023B10564003).

## Disclosure

All authors have made substantial contributions to the work.

## Ethics Statement

All animal experiments were approved by the Ethics Committee for Animal Experiments of the South China Agricultural University (Ethics Committee Approval Number 2023C089) and conducted in compliance with the Regulations for the Administration of Affairs Concerning Experimental Animals approved by the State Council of the People’s Republic of China.

## Conflicts of Interest

The authors declare no conflicts of interest.

## Supporting Information

Additional supporting information can be found online in the Supporting Information section.

## Supporting information


**Supporting Information** Figure S1 displays the original immunofluorescence images corresponding to the quantitative analysis results presented in Figure 2. These representative microscopic images show the intuitive changes of RABV‐positive fluorescence foci in NA and BHK‐21 cells infected with CVS‐11 or HEP‐Flury after treatment with gradient concentrations of andrographolide. All supporting images were acquired under consistent experimental conditions and provide direct visual evidence to verify the dose‐dependent inhibitory effect of andrographolide on RABV replication. No supporting tables are included in this study.

## Data Availability

All relevant data supporting the findings of this study are openly available in ScienceDB at DOI: https://doi.org/10.57760/sciencedb.28630 under the terms of the Creative Commons Attribution 4.0 International License (CC‐BY 4.0). This license permits unrestricted use, distribution, and reproduction in any medium, provided that the original authors and source are credited.
