# Supplementary material for: Andrographolide Inhibits Rabies Virus Replication
Source: Transbound Emerg Dis. 2026 Sep 22;2026:8934817. doi: 10.1155/tbed/8934817 (PMC13596000; doi:10.1155/tbed/8934817)
Supplement: Supplementary file 1 [file TBED-2026-8934817-s001.doc]

**Figure S1**

Figure S1 Immunofluorescence assay of Andro inhibiting RABV

(A) Inhibitory effect of Andro on CVS-11 in NA cells; (B) Inhibitory effect of Andro on HEP-Flury in NA cells; (C)Inhibitory effect of Andro on CVS-11 in BHK cells; (D) Inhibitory effect of Andro on HEP-Flury in BHK cells.
